# Acute effects of vibration foam rolling and local vibration during warm-up on athletic performance in tennis players

**DOI:** 10.1371/journal.pone.0268515

**Published:** 2022-05-18

**Authors:** Feng Wang, Zhihua Zhang, Chengliang Li, Delong Zhu, Yueying Hu, Honghao Fu, Huan Zhai, Yongjun Wang

**Affiliations:** 1 School of Physical Education, Huazhong University of Science and Technology, Hubei, China; 2 The College of Humanities and Law, Beijing University of Chemical Technology, Beijing, China; 3 Shenyang Sport University, Liaoning, China; 4 Shanghai Sport University, Shanghai, China; Federation University Australia, AUSTRALIA

## Abstract

Athletes are currently fond of vibration foam rollers (VFRs) and commercial portable vibration percussion devices (PVPDs). It is still unknown whether using these devices during warm-up has an immediate impact on athletic performance. A randomized block design was used in this study. The acute effects of VFR and PVPD on tennis players’ athletic performance during warm-up were compared. For the countermovement jump (CMJ), reactive strength index (RSI), and hexagon test (HT), the difference in performance between all interventions was significant (p = 0.007–0.034, η^2^_p_ = 0.266–0.364). Only those who received VFR had significantly different CMJ and HT results when compared to the control group (CMJ height = 53.18 ±4.49 cm, p = 0.03, d = 1.26; HT time = 10.73 ±0.4 s, p = 0.03, d = 1.12). Participants’ RSI values were significantly different after VFR (RSI = 2.01 ±0.11 cm·mm^–1^, p = 0.012, d = 1.76) and PVPD (RSI = 1.99 ±0.11 cm·mm^–1^, p = 0.025, d = 1.52) compared to the control group. Therefore, when using VFR and PVPD as part of warm-up protocols for tennis players of varying skill levels, VFR could have an immediate positive effect on power, reactive strength, and change of direction performance, while PVPD could immediately improve reactive strength performance.

## Introduction

Tennis is defined by intermittent high-intensity and short movements [1, 2], accelerations and decelerations [1, 2], change of direction (COD), and running at varying speeds with moderate-to-long rest periods [3]. Within a 2.5 m radius of a player’s ready position, 80% of all strokes are played [3]. Furthermore, players can make over 1,000 directional changes during a match [4]. Players are widely accepted to require high levels of physical fitness, including power, strength, COD, acceleration, dynamic balance, and a highly developed neuromuscular coordination ability, to execute difficult shots and compete effectively against skilled opponents [5, 6].

Warming up is a common ritual performed by most athletes prior to nearly every athletic event in order to induce the body into a suitable state for achieving optimum performance [7–9]. Warm-up is commonly used to raise muscle and body temperature, improve blood circulation, improve performance, and prevent injury [8, 10]. New warm-up modalities have been investigated in recent years in order to reduce monotony and improve performance. Self-myofascial release (SMR) is a type of warm-up regimen that is also known as ’foam rolling’ (FR) or ’self-myofascial release’ (SMR) [11, 12]. One of the most popular SMR tools is the foam roller [13], and individuals simply use their body weight to apply pressure on target soft tissues over the foam roller to create friction. Many researchers have investigated the acute effects of increasing joint range of motion [14] and muscle flexibility [10], and reducing muscle stiffness [15] without a concomitant decrease in subsequent performance such as sprint time, jump height [12], and muscle strength [16]. Some studies even discovered that FR had an immediate positive effect on athletic performance, such as strength and speed [17].

Vibration is a mechanical stimulus characterized by an oscillatory wave [18] that has potential applications in exercise performance [19]. There are two types of vibration training: whole-body vibration (WBV) and local vibration [20] or focal vibration [21]. Vibration exercises can also be incorporated into or used as a stand-alone warm-up modality [8]. When compared to WBV [19], local vibration, where the vibration stimulus is applied directly to a specific muscle group or body segment [22], is well-tolerated, cost-effective, and portable. When used as part of warm-up protocols, local vibration has been shown to have an immediate effect on increasing muscle activity and metabolic response [19, 23]. Previous studies used a variety of portable local vibrators, including strapped-vibrators [24], hand-held vibrating dumbbells [22], and vibrating cables [25]. Some studies, for example, found that using local vibration devices during warm-up had an immediate effect on quadriceps function [26], power [27], hamstring and quadriceps flexibility [28]. Recently, the majority of athletes and rehabilitation specialists have favored a commercial manual local vibration device known as portable vibrational percussion devices (PVPD) for its high efficiency and portability. However, no research has been done on its use and effect in warm-up regimens. In warm-up programs, a vibrating foam roller (VFR) combining foam rolling techniques with local vibration has also been used to improve athletic performance [29]. Several studies have confirmed the acute effect of VFR on joint ROM [29], perceived joint stability [30], an individual’s pain tolerance [31], muscle strength [13], COD [7], power [29], dynamic balance [13], and so on during warm-up. These studies compared vibrating foam rollers to non-vibrating foam rollers [29, 30] as well as the stretching method [14]. Currently, no study has compared the acute effects of VFR and PVPD on athletic performance during warm-up routines.

Therefore, the primary goal of this study was to see if using VFR and PVPD as part of a warm-up routine had any acute effects on COD, dynamic balance, power, and reactional strength in tennis players, and if so, whether there was a significant difference in the acute effects of these two devices. It was hypothesized that both VFR and PVPD would immediately improve tennis players’ athletic performance, and significant differences in the acute effects on athletic performance between VFR and PVPD were also anticipated.

## Materials & methods

### Participants

Twenty-seven male tennis players (age: 20.4 ±1.3 y, body mass: 71.6 ±7.8 kg, height: 1.81 ±0.63 m, tennis experience: 10 ±0.7y) from the tennis team of Beijing University of Chemical Technology participated. This team had a high ranking among the college tennis teams in China. Participants were free from cardiovascular diseases, musculoskeletal or traumatic injuries and other diseases that could limit normal physical activity. The individual in this manuscript has given written informed consent (as outlined in PLOS consent form) to publish these case details. This study was performed according to the latest version of the Declaration of Helsinki, and the experimental protocol was approved by the College of Humanities and Law of the university.

### Study design

**Fig 1:**
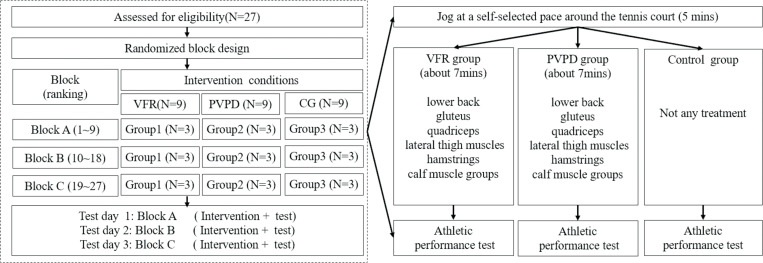
Trial procedures.

A randomized block design was used. Participants were divided into three blocks according to their latest team ranking provided by the coach. Each block included nine participants: Block A (ranking 1 to 9), Block B (ranking 10 to 18), Block C (ranking19 to 27). Each block was further divided into three groups randomly, which were randomly assigned to the VFR group, PVPD group, and control groups (CG). Each group had three participants who had just received corresponding treatment randomly (Fig 1).

The experiment was performed on outdoor tennis courts at Beijing University of Chemical Technology. Before the experimental day, participants were given a familiarization session, in which they were familiarized with the experimental process and all interventions. After 24 h of the familiarization session, the participants performed the intervention under the guidance of a certified physical therapist. Immediately, power, reactive strength, lateral acceleration, COD, and dynamic balance were measured sequentially after each intervention. An operator guided each test and verbally reminded the participants to take the athletic performance test as quickly as possible. The participants in Block A, Block B and Block C received intervention and test on independent experimental days, and the experiments were carried out for three consecutive days. The experiment was started at 9:00 am, and the temperature was about 24°C. The participants were asked to wear tennis shoes to participate in the experiment and avoid excessive physical activities 48 h before the test.

### Experimental intervention

The participants in CG just jogged for 5 mins at a self-selected pace [32] and then performed each test directly.

**Fig 2:**
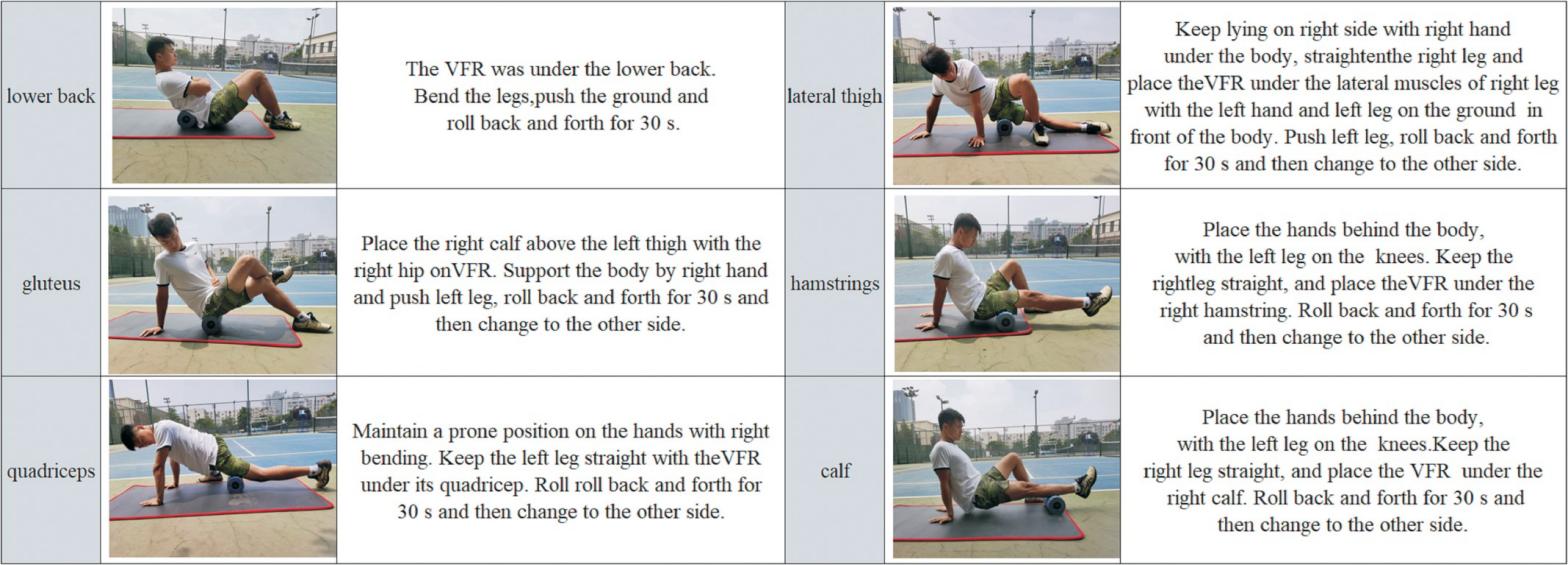
VFR protocols.

In the VFR group, a commercial vibrating foam roller (dimensions: 29.8 × 14 × 14 cm^3^; weight: 1.6 kg, VYPER2.0®, Hyperice, United States) was used, and the vibrational frequency was set to 60Hz [21]. The participants first jogged for 5 mins at a self-selected pace [32], then VFR was performed sequentially on the lower back, gluteus, quadriceps, lateral thigh muscles, hamstrings, and calf muscle groups [33]. The participants put the target muscle groups on the roller to perform VFR (Fig 2). The lower back muscles were rolled bilaterally, while other muscle groups were rolled unilaterally. For lower limbs, VFR was performed first on the right limb followed by the left limb. The entire length of the muscle was covered from origin to insertion [17]. The participants were asked to roll back and forth for the 30s at 40 beats per minute by using a metronome [34]. The participants performed one set of VFR on each muscle group with no interval between each VFR. Each muscle group received the 30s of VFR, and the entire process of VFR took about 7 mins. The protocols for these interventions are detailed in Fig 2.

**Fig 3:**
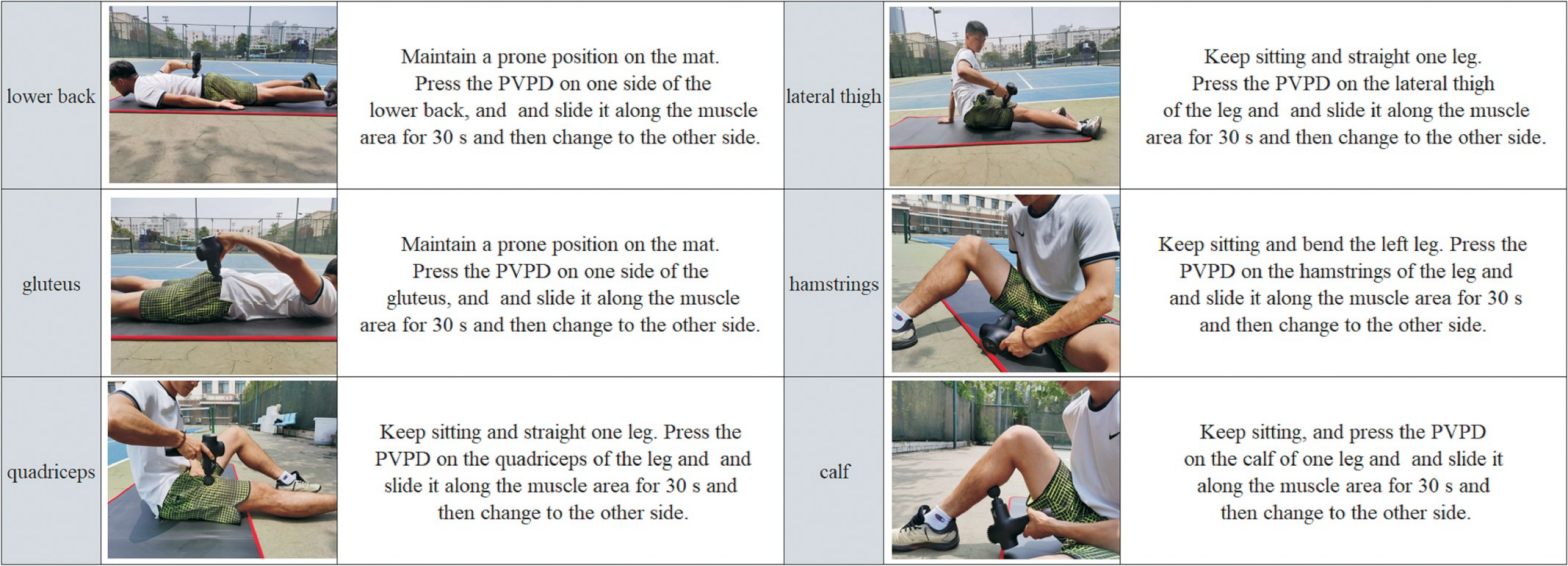
PVPD protocols.

In the PVPD group, a portable vibrational percussion device (dimensions: 26 × 17 × 6.5 cm^3^; weight: 0.9 kg, therapy head: 5, OUTSO, China) was used, and the vibrational frequency was set to 60Hz [35]. The spherical therapy head was selected for all target muscle groups. The participants firstly jogged for 5 mins at a self-selected pace [32], then PVPD was performed sequentially on the lower back, gluteus, quadriceps, lateral thigh muscles, hamstrings, and calf muscle groups. The participants performed the PVPD by holding the handle of the device to press the therapy head on the target muscle group and slide it along the muscle area for 30 s [36]. The entire length of the muscle was covered from the origin to insertion [17]. All the muscles were vibrated unilaterally, and each muscle group received the 30s of PVPD, and the entire process of PVPD took about 7 mins. The protocols for these interventions are detailed in Fig 3.

### Measures

After receiving the VFR or PVPD, the participants immediately performed the countermovement jump (CMJ) test, drop jump test、hexagon test、2.5 m lateral acceleration test, Y- balance test in order. The interval between each test was 3 mins.

#### CMJ test

CMJ test that is reliable for assessing the power of the lower limb [29] was selected. The Smart Jump (Fusion sport, AUS) was used for the test. It consisted of a jump mat and a control unit connected to the jump mat for calculating the height. When performing CMJ, participants stood on the jump mat with their feet shoulder-width apart and hands on the hips. The participants then squatted down until the knees were bent at 90°, then immediately jumped as high as possible, and kept knees straight during the flight phase, landing back on the mat on both feet at the same time. They were allowed to jump three times with 1 min of rest between each jump, and two trial jumps were given. The highest jump was selected for evaluating the performance of the CMJ. In this study, the intraclass correlation coefficient (ICC) was 0.978.

#### Drop jump test

Smart Jump was employed. A tennis-specific movement pattern during the starting movement was the split-step based on a stretch-shortening cycle (SSC) [35]. The reactive strength index (RSI) was a useful parameter for quantifying the SSC when evaluating the drop jump [37]. Therefore, the RSI was measured in this study. The drop jump was performed onto the jump mat from a 30 cm^3^ wooden jump box [14]. Participants began by standing on the box, taking a step forward with their left leg, and landing both feet on the mat. After landing, participants must jump as high as they can in the shortest amount of time. Throughout the procedure, they were instructed to keep their hands on their iliac crest. The RSI would be calculated automatically by the control unit by dividing the jump height by the contact time. Participants were allowed to jump three times with one minute of rest between each jump, and they were given two trial jumps. The highest RSI value was used in the analysis. The ICC was calculated to be 0.859.

#### 2.5 m lateral acceleration test (2.5m LAT)

**Fig 4:**
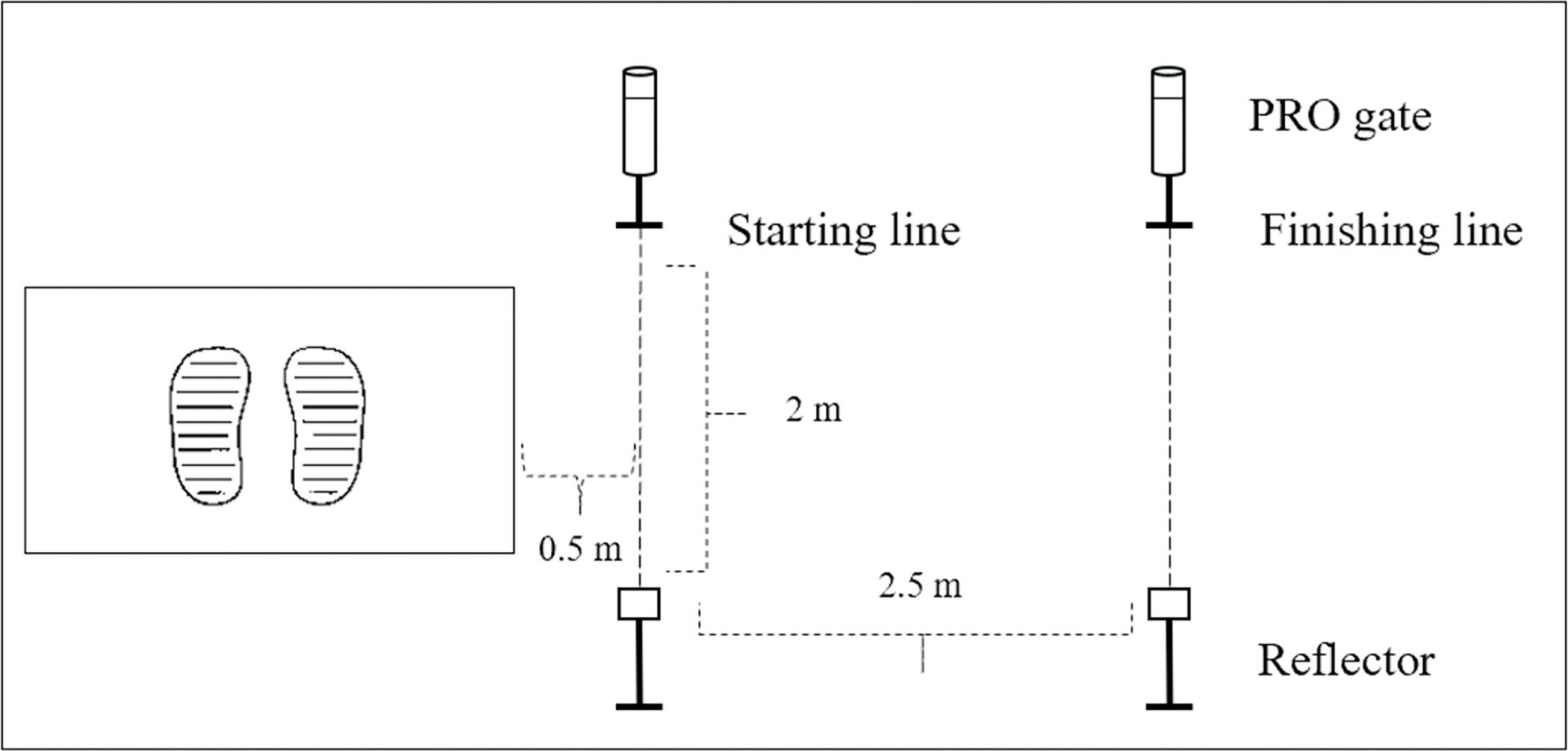
The 2.5 m LAT chart.

Since 80% of all strokes were played within 2.5 m of the player’s ready position [38], and the lateral movement accounted for more than 70% of all the movement in a match [39], so it was feasible to use 2.5 m as the length of lateral acceleration test [40]. The Smart Speed Timing Gate System (Smart Speed PRO V1, Fusion Sport, Australia) and Smart Jump were used to measure 2.5 m LAT. The system contained two PRO gates, two reflectors, and one Smart HUB for data receiving and processing. The infrared ray from the first PRO gate to its corresponding reflector was considered as the starting line, which was 1 m above the ground. The other infrared ray was considered as the finish line, which was 2.5 m from the starting line and 1 m above the ground. The distance between the jump mat and the starting line was 0.5 m [41]. The distance between each PRO gate and the reflector was 2 m (Fig 4). Reactive acceleration mode was selected.

As the first starting step, the gravity step was shown to be the fastest step to move laterally [42]. Thus participants were asked to use the gravity step as the first step while holding their tennis racket. The right side LAT was performed first, followed by the left side. Participants were given one trial acceleration for each side, then LAT on each side was performed three times with 1 min interval between each LAT. The best score was considered for the study. The ICCs of the right and left side LAT were 0.965 and 0.924, respectively.

#### Hexagon test

The hexagon test (HT) was a reliable and effective tool for assessing COD (ICC = 0.924–0.938) [43]. On the tennis court, a hexagon with 24-inch sides and 120° angles was marked with white tape (length: 24 inches, width: 1 inch) [44]. A stopwatch was used to record the time to the nearest 0.01 s. Participants stood in the center of the hexagon, feet together, facing forward. Participants began jumping over each side and back to the center in a clockwise fashion after hearing the command "Go" and simultaneously turning on the stopwatch. Participants were required to complete three laps as quickly as possible. The stopwatch was turned off when they returned to the center after the final lap. Participants were instructed to face forward at all times during the test. The test could be restarted if they stepped on any side or jumped over the wrong side. They were allowed to jump three times with one minute of rest between each jump, and they were given two trial jumps. For the analysis, the best score was used. The ICC was determined to be 0.935.

#### Dynamic balance test

The Y-Balance Test (YBT) has been demonstrated to be reliable in determining dynamic balance [45]. Thus, in this study, YBT was used to assess participants’ dynamic balance. The Y-Balance Test Kit^TM^ (Move2Perform, Evansville, IN) was used, and the test was carried out in accordance with previous research [45]. The participants’ leg length from the anterior superior iliac spine to the center of the medial malleolus [46] was first measured with tape. Participants were then instructed to stand barefoot on one leg in the center of the device and use the free limb to push the target as far as possible in various directions, including anterior, posteromedial, and posterolateral. They pushed the target with their right leg first (as the free leg), and then with their left leg. Participants were given one trial before being required to push the target three times in each direction with no rest in between. The greatest distance traveled was recorded. The test score was calculated as follows: [anterior distance (cm) + posteromedial distance (cm) + posterolateral distance (cm)]/3 × length of leg(cm) × 100. If participants were unable to maintain a one-legged stance, touch the target with their toes, or withdraw the free leg to the ready position during the test, the test was restarted. The right and left sides’ ICCs were 0.958 and 0.988, respectively.

### Statistical analyses

The data were analyzed using IBM SPSS Statistics ver. 20.0 (Chicago, IL, USA). The data were presented as the mean ±standard deviation (SD). The Shapiro–Wilk and Levene tests were used to determine normality and variance homogeneity, and the results of these two tests were all greater than 0.5 in this study. Data were analyzed by Two-way ANOVA with post hoc Bonferroni tests. To investigate the effects of different conditions on dependent variables, Effect size (ES) was classified as small (0.01 ≤ η^2^_p_ < 0.06), medium (0.06 ≤ η^2^_p_ < 0.14) or large (η^2^_p_ ≥ 0.14) according to Cohen’s η^2^_p_ during the Two-way ANOVA. The ES was classified as trivial (d ≤ 0.19), small (0.20 ≤ d ≤ 0.49), medium (0.50 ≤ d ≤ 0.79) or large (d ≥ 0.80) according to Cohen’s during the post hoc Bonferroni test. The test-retest reliability was assessed using the intraclass correlation coefficient (ICC). The level of significance was set at p < 0.05.

## Results

### CMJ height outcomes

For CMJ height (Table 1), Two-way ANOVA showed that there were significant differences between the effects of the interventions (p = 0.033, η^2^_p_ = 0.266), but no significant difference between blocks (p = 1.79, η^2^_p_ = 1.45). Post hoc pairwise comparisons between VFR and CG showed that participants in VFR had the greatest CMJ height (CMJ height = 53.18 ±4.49 cm) and exhibited significant differences (p = 0.03, d = 1.26) compared to the CMJ height of participants in CG (CMJ height = 47.92 ±3.82 cm). No significant difference was found between the VFR and PVPD (CMJ height = 50.08 ±3.97cm) or between PVPD and CG (p > 0.05).

**Table 1:** Descriptive results of ANOVA.

Test	Intervention				Test of Between-Subjects Effects					
	VFR	PVPD	CG	ICC	Block			Intervention		
					F_0_	P_0_	η^2^_p0_	F_1_	P_1_	η^2^_p1_
CMJ(cm)	53.18±4.49	50.08±3.97	47.92±3.82*	0.978	1.861	0.179	0.145	3.996	0.033	0.266
RSI(cm·mm^-1^)	2.01±0.11	1.99±0.11	1.86±0.05#	0.859	0.189	0.829	0.017	6.297	0.007	0.364
HT(s)	10.73±0.4	11.02±0.45	11.39±0.73 *	0.935	3.426	0.051	0.237	3.977	0.034	0.266
LAT_L_(s)	0.940±0.098	1.004±0.138	0.951±0.090	0.924	2.126	0.143	0.162	0.933	0.409	0.078
LAT_R_(s)	0.896±0.100	0.967±0.107	0.954±0.122	0.965	0.141	0.869	0.013	0.99	0.387	0.083
YBT_L_	0.879±0.081	0.849±0.074	0.872±0.036	0.988	1.387	0.271	0.112	0.514	0.605	0.045
YBT_R_	0.876±0.123	0.867±0.085	0.878±0.064	0.958	0.96	0.398	0.08	0.794	0.465	0.067

Note: HT, Hexagon test; YBT_L_, YBT of left leg; YBT_R_, YBT of right leg; LAT_L_, 2.5 m lateral acceleration to left side; LAT_R_, 2.5 m lateral acceleration to the right side. Block result: F_0_, P_0_, η^2^_p0_. Intervention result: F_1_, P_1_, η^2^_p1_.

*Significant difference (p < 0.05) compared with the VFR

# Significant difference (p < 0.05) compared with both VFR and PVPD. ICC, the reliability of each test.

### RSI outcomes

For RSI (Table 1), Two-way ANOVA showed that there were significant differences between the effects of the interventions (p = 0.007, η^2^_p_ = 0.364), but no significant difference between blocks (p = 0.829, η^2^_p_ = 0.17). CG had the lowest RSI (1.86 ±0.05 cm·mm^–1^), VFR (RSI = 2.01 ±0.11 cm·mm^–1^, p = 0.012, d = 1.76), and PVPD (RSI = 1.99 ±0.11 cm·mm^–1^, p = 0.025, d = 1.52) showed significantly higher RSI than that in CG. No significant difference between the RSI for VFR and PVPD was found according to the post hoc test results (p > 0.05).

### HT outcomes

For HT (Table 1), Two-way ANOVA showed significant differences between the effects of the interventions (p = 0.034, η^2^_p =_ 0.266), but no significant difference was found between blocks (p = 0.51, η^2^_p =_ 0.237). Post hoc pairwise comparisons between VFR and CG showed that HT in VFR exhibited significantly lower time (HT = 10.73 ±0.4 s, p = 0.03, d = 1.12) compared to that in CG (HT = 11.39 ±0.73s), but no significant differences between VFR and PVPD (HT = 11.02 ±0.45 s) or between PVPD and CG were found (p > 0.05).

### Other test outcomes

For the YBT and the 2.5 m LAT, no significant differences were found for the effects of interventions (p > 0.05) or blocks (p > 0.05) in Two-way ANOVA (Table 1).

## Discussion

This was the first study to compare the acute effects of VFR and PVPD as a warm-up protocol on the athletic performance of tennis players, including COD, reactive strength, power, dynamic balance, and lateral acceleration. The main findings revealed no difference in the acute effect of VFR and PVPD on tennis players’ athletic performance. Nonetheless, the VFR had a significant positive effect on the reactive strength, COD, and power of the lower limbs of tennis players of various skill levels. The PVPD simply improved the reactive strength performance right away. Neither of these devices had a significant positive effect on dynamic balance or 2.5 m lateral acceleration.

**Fig 5:**
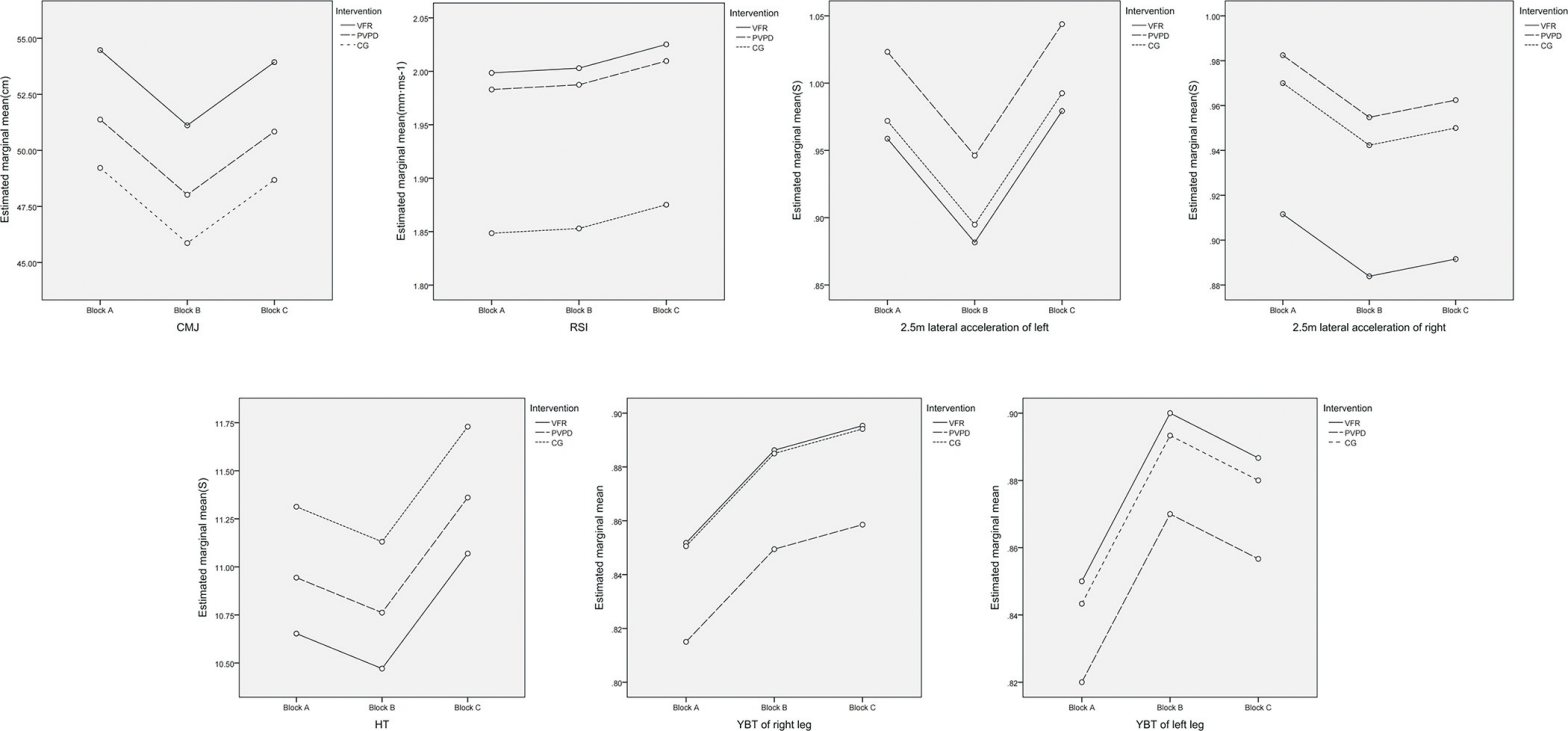
The estimated marginal mean profile plots.

Contrary to our hypothesis, the results showed no difference in the acute effect of VFR and PVPD on any athletic performance of tennis players. In theory, VFR combined the dual effects of local vibration and foam rolling, whereas PVPD only used local vibration; when the vibration frequency was the same (60Hz), the acute effect of VFR on athletic performance should be significantly greater than that of PVPD. This pattern could also be seen in the estimated marginal mean profile plots (Fig 5). The participants in this study only did one set of VFR and PVPD on each muscle group. This may decrease the difference between the two interventions differ.

VFR’s CMJ height was significantly higher than CG’s. This finding contradicted the findings of most previous studies, which concluded that SMR had no positive effect on lower extremity power [10, 14, 47–49]. Lim et al., for example, demonstrated that the FR, with (38 Hz, 60 s × 5 sets) or without vibration (60 s × 5 sets), could not improve vertical jump height in college students between pre- and post-treatment (p > 0.05), with no significant difference between groups (p = 0.887) [10]. Unfortunately, no studies on the acute effects of local vibration on CMJ, particularly using PVPD as a local vibration tool, were found. However, there have been some studies on the acute effects of other local vibration devices on upper extremity power [25, 27]. The results of these studies all showed that the power of the biceps increased significantly during bilateral biceps stimulation compared to the nonvibratory stimulation group (p <0.05) [25, 27]. Therefore, to some extent, our PVPD research was a supplement to previous studies.

The acute effects of VFR in this study could be explained by the following mechanism. The mechanical vibration produced by the VFR activated proprioceptors, particularly muscle spindles at the muscle belly [50], which produced Ia afferent signals to activate large α-motoneurons, potentially recruiting more motor units [51]. Meanwhile, the local vibrations induced the tonic vibration reflex in the muscles [52]. This reflex activated sensory nerve fibers, which increased neuromuscular activity [53] and motor unit recruitment via muscle spindles [54]. The friction between vibrating tissues and the foam roller and the body causes an increase in local muscle temperature when participants perform VFR [55]. The post-vibratory effects were aided by increased blood flow [56]. Furthermore, the vibration frequencies used in our study (60 Hz) were higher than those used in the previous study. This frequency was closer to the optimal frequency for the strongest activation of Ia muscle afferents (75 Hz) [21].

There was no statistically significant difference in CMJ height between PVPD and CG. In our experiment, we used a frequency of 60 Hz. Although this frequency was high, it was insufficient to distinguish between PVPD and CG in CMJ height. In future studies, a higher frequency may be considered.

In this study, the RSI in VFR and PVPD was significantly higher than in CG. Both devices were said to have an immediate positive effect on the tennis player’s reactive strength. There have been few studies on the acute effects of VFR or local vibration exercises on reactive strength. Nonetheless, more research on the acute effects of FR or the combination of FR and dynamic stretching (DS) on reactive strength has been conducted. However, those studies concluded that using FR [57, 58] or DS+FR [14, 59] during warm-up did not immediately improve or degrade reactive strength performance when compared to CG. For example, Grabow et al. discovered that when FR (3 sets × 60 s) was performed at different intensities, there was no significant difference in height, contact time, or performance index of drop jump, regardless of the main effects of time (p = 0.068) and intensity (p = 0.249), or the interaction between time and intensity (p = 0.420) [57]. In contrast to previous studies, ours used a novel intervention device. A VFR combining local vibration and self-fascia relaxation was used, which may have provided more stimulation than a simple FR. Furthermore, the use of PVPD to generate local vibration demonstrates its effectiveness in improving RSI. Drop jump was found to be associated with a fast stretch-shortening cycle (SSC) [60]. Stretch-reflexes, according to Komi, played a significant role in SSC and contributed to force generation during the eccentric phase [61]. The role of muscle spindles in improving CMJ performance during VFR was discussed above, and it was also applicable to SSC. Local vibration or FR was reported to cause soft tissue deformation and activate the muscle spindle, which strengthened the function of the SSC [62]. As previously demonstrated, PVPD had no immediate positive effect on CMJ involving slow SSC [60]. The findings also revealed that both VFR and PVPD had an immediate positive effect on reactive strength involving fast SSC.

Only the HT in VFR had a significant difference with CG in our study. Some studies found no immediate benefit from FR or FR+DS on COD performance in a 5–10–5 shuttle run [63] or T-test [59]. Other studies, however, had found an immediate effect of VFR, FR, or a combination of VFR and other interventions on COD [34, 64], despite the fact that the COD tests used in those studies differed from the COD test used in our study. Lyu et al. discovered that both VFR (28 Hz, posterior calf muscles, 1 set × 30 s) and DVR (dynamic muscle contraction + VFR) improved COD performance (in the figure-of-8 hop test) by 1.98% and 2.53%, respectively, when compared to pretest results [34]. COD is defined as a rapid whole-body movement with a change in velocity or direction in response to a stimulus [65], and it is associated with leg muscle strength, power, and reactive strength [65]. Reactive strength, in particular, was a good predictor of COD [66]. Pearson’s correlation was also used in our study to determine the relationship between COD, CMJ, and RSI. In the VFR group, there was a strong negative correlation between HT and RSI (r = –0.702, p = 0.035) and between the hexagon test and CMJ (r = –0.675, p = 0.046). As a result of VFR’s significant acute effects on CMJ and RSI, as well as a strong correlation between them, we can explain why VFR produced significant acute effects on COD.

Furthermore, PVPD had no significant positive effect on HT in this study. We also found no research on the immediate effect of local vibration on COD. Therefore, more comprehensive research is required in the future.

In comparison to CG, VFR and PVPD have no significant effect on the 2.5 m LAT. Some studies [59, 67] found that FR, VFR, or WBV had no immediate effect on sprint or acceleration performance, which was similar to our findings. According to Cochrane et al., the reason vibration exercises could not improve sprint performance could be due to the complex and dynamic nature of sprinting, which could result in the loss of the increased power gained in vibration exercises [8]. The 2.5 m LAT was not the same as the short distance sprint. It consisted of a series of unique tennis movements such as the standing prepare posture, split-step, body turning, and accelerating toward a target. These movements may interfere with the enhanced ability gained from the VFR or PVPD. Furthermore, 2.5 m may be too short to demonstrate the effect of the interventions on lateral acceleration.

The results also revealed that there was no statistically significant difference in YBT between all interventions. This finding contradicted previous research that found VFR and FR to have a positive effect on dynamic balance [17, 34]. 6 minutes of VFR (28Hz, 3 sets × 30 s) and FR on quadriceps and hamstrings of both legs, for example, were found to significantly increase YBT compared to CG (p <0.01) [17]. Although no studies on the effect of PVPD on dynamic balance were found, it was reported that the WBV could immediately improve subjects’ dynamic balance [68]. The improvement in dynamic balance could be attributed to improvements in joint stability [30], joint range of motion [11, 20, 29], and muscle flexibility [10, 30] following VFR or FR. However, in our study, VFR or PVPD had no positive effect on the YBT of the tennis players, despite the fact that the YBT in VFR and PVPD appeared to be higher than that in CG based on the profile plots (Fig 5). Because the YBT was administered relatively late in the study, the effects of the two interventions were most likely weakened. If YBT was performed immediately after VFR and PVPD, the results could be different. Therefore, additional research may be required in the future.

There were no differences in performance between the blocks, according to the results. This meant that the effects of all interventions on athletic performance were independent of the participants’ skill level. Therefore, the findings suggested that VFR and PVPD were appropriate for tennis players of varying skill levels.

### Limitations

To begin, our study’s participants were semi-amateur tennis players. The results might have been different if the experiments had been conducted with higher-level tennis players. Second, because several tests were conducted, the immediate effects of the interventions may have weakened in subsequent tests. Third, VFR and PVPD were only available at 60 Hz, with no other frequencies used. Furthermore, manual timing in some tests may be inaccurate.

## Conclusion

During warm-up, when using VFR and PVPD as part of warm-up protocols for tennis players of various skill levels, the VFR could have an acute positive effect on the performance of reactive strength, COD, and power of the lower limbs of tennis players of various skill levels. The PVPD could simply improve reactive strength performance right away. Neither of these devices significantly improved dynamic balance and short-distance lateral acceleration performance. The acute effect of these devices on any athletic performance was the same.

## Abbreviations

VFR: Vibration foam roller
PVPD: Portable vibration percussion device
CG: Control group
CMJ: Countermovement jump
2.5 m LAT: 2.5 m lateral acceleration
HT: Hexagon test
YBT: Y-balance test
RSI: Reactive strength index
WBV: Whole-body vibration
ROM: Range of motion
ICC: Intraclass correlation coefficient
SMR: Self-myofascial release
SSC: Stretch-shortening cycle
SD: Standard deviation
NVFR: Non-vibration foam rolling
DS: Dynamic stretching
DVR: Dynamic muscle contraction + VFR treatments
